# Potassium Management for Improving Growth and Grain Yield of Maize (*Zea mays* L.) under Moisture Stress Condition

**DOI:** 10.1038/srep34627

**Published:** 2016-10-03

**Authors:** Amanullah, Asif Iqbal, Irfanullah, Zeeshan Hidayat

**Affiliations:** 1 Department of Agronomy, Faculty of Crop Production Sciences, The University of Agriculture, Peshawar-25130 Pakistan

## Abstract

Potassium (K) fertilizer management is beneficial for improving growth, yield and yield components of field crops under moisture stress condition in semiarid climates. Field experiments were conducted to study the response of maize (*Zea mays* L., cv. Azam) to foliar and soil applied K during summer 2013 and 2014. The experiments were carried out at the Agronomy Research Farm of The University of Agriculture Peshawar, Northwest Pakistan under limited irrigation (moisture stress) condition. It was concluded from the results that application of foliar K at the rate of 1–3% and foliar Zn at the rate of 0.1–0.2% was more beneficial in terms of better growth, higher yield and yield components of maize under moisture stress condition. Early spray (vegetative stage) resulted in better growth and higher yield than late spray (reproductive stage). Soil K treated plots (rest) plots performed better than control (K not applied) in terms of improved growth, higher yield and yield components of maize crop. The results further demonstrated that increasing the rate of soil applied K up to 90 kg P ha^−1^ in two equal splits (50% each at sowing and knee height) improve growth and maize productivity under semiarid climates.

Maize (*Zea mays* L.) is the third most important cereal crop in Pakistan after wheat and rice. In Northwest Pakistan (Khyber Pakhtunkhwa), maize ranked 2^nd^ after wheat in its importance. During 2012, maize was cultivated on an area of 1087.3 thousand hectares with the total production of 4338.3 thousand tons and national average yield of 3990 kg ha^−1^ in Pakistan. In Khyber Pakhtunkhwa province, maize was grown on 475.3 thousand hectares area with a total production of 887.8 thousand tones with very low and average yield of 1868 (kg ha^−1^)^1^. According to ref. 2, the two major reasons of low maize productivity under semiarid condition are: (1) imbalanced use of chemical fertilizers and (2) water stress (dryland) condition. The two key features which adversely affect crop production under semiarid climates are the low amounts of rainfall and the unreliability^3^. Crop growth and productivity decline when plants suffer from drought stress in their life cycle^4^,^5^,^6^,^7^,^8^.

The growers in the drylands do not apply potassium (K) fertilizers and therefore the crops not only suffer from drought stress but also suffer from K deficiency. In Pakistan, K status of soils is rapidly decreasing at the painful rate. The net K exhausting rate is even steeper 0.3 kg ha^−1^ year^−1^. This may be due to the trifling (0.8 kg ha^−1^ year^−1^) use of K in Pakistan as compared to world average K use (15.1 kg ha^−1^ year^−1^)^9^. In fact, today the use of K rate is almost half of what it was in mid 90s. In other words the current usage of K is almost 25% of what the government target was at the end of 8^th^ Five-year Plan^10^. Under K deficient conditions photosynthesis in crops is depressed as a consequence of sucrose accumulation in the leaves and its negative effect on gene expression^11^,^12^.

Balanced crop nutrients management is a key factor for improving crop productivity and growers income under semiarid climates^8^,^13^,^14^. The published research work of many workers indicates that K application under moisture stress condition not only improve crop tolerance to drought stress but also improve crop growth, dry matter partitioning and increase yields significantly^15^,^16^,^17^,^18^. Potassium has important functions in plant water relations where it regulates ionic balances within cells and also play a significant role in the activation of more than 60 enzymes which catalyze various metabolic process^19^ and uptake and translocation of nitrates from root to aerial parts of plants^20^.

The problem of water scarcity and unavailability of nutrients to the crops under semiarid climates can be overcome by the application of foliar nutrition^2^,^21^,^22^. Foliar nutrition under semiarid climates not only applies nutrients but is also beneficial in terms of providing water to the crops^2^. Foliar application of nutrients is in general helpful to satisfy plant requirement and also has a high efficiency^23^. Foliar K application is particularly well adapted to this form of fertilization because soon after foliar spraying takes place it is rapidly translocated from the leaves^24^. Foliar K application is thus an attractive means especially in arid zones where a lack of water under low rainfall conditions in summer drastically depresses absorption of soil nutrients^25^,^26^ reported that foliar application of K at grain filling stage of wheat alleviated the adverse effect of water deficit and increased the yield and yield components tremendously.

This objectives of this research projects were: (1) to find out suitable soil applied K level and its application time and (2) to find out suitable foliar applied K level and its application time for increasing maize productivity under limited irrigation (moisture stress) condition.

## Materials and Methods

### Site description

Field experiments were conducted at the Agronomy Research Farm of The University, of Agriculture Peshawar, Pakistan for two years in summer growing seasons (July–October) 2013 (Y1) and 2014 (Y2). The research farm is located at 34.01°N, 71.35°E, at an altitude of 350 m above sea level in the Peshawar valley. Peshawar is located about 1600 km north of the Indian Ocean and has semiarid climate. The research farm is irrigated by the Warsak canal from the Kabul River. Earlier^13^ reported that soil of the farm is clay loam in texture, alkaline (pH 8.2), and calcareous^27^, with a low organic matter content of 8.7 (g kg^−1^)^28^ and low concentrations of extractable phosphorus of 6.57 mg kg^−1^ and exchangeable potassium (AB-DTPA) of 121 (mg kg^−1^)^29^. The climate of the area is semiarid where the mean annual rainfall is very low (300 to 500 mm), 60–70% rainfall occurs in summer, while the remaining 30–40% rainfall occurs in winter^30^.

### Experimentation

To study the effect of foliar potassium (K) and zinc (Zn) levels on growth and yield of maize under moisture stress condition, field experiments were conducted at the Agronomy Research Farm, The University of Agriculture Peshawar, Pakistan, during summer 2013 and summer 2014. The experiment in each year (year one and year two) was laid out in a randomized complete block design having three replications. Each replication was consisted of 20 treatments having three foliar K levels (K1 = 1, K2 = 2 and K3 = 3% foliar K), three foliar Zn levels (Zn1 = 0.1, Zn2 = 0.2 and Zn3 = 0.3% foliar Zn), one control (no K and Zn spray) was used and two application times [T1 = at V12 vegetative growth stage (ear shoots were formed just before tassel formation) and T2 = at R1 reproductive growth (silks were visible outside the husks)]. The sources used for foliar K and Zn were Potassium Helb (36% K) and Zinger (10% Zn), respectively, and foliar spray was applied in the morning. Plot size of 4.5 m × 4 m (6 rows, 4 m long and 75 cm apart) was used. A recommended dose of 120 kg N ha^−1^ (urea) and 60 kg P ha^−1^ (Di Ammonium Phosphate) was applied uniformly to all plots along with control. The required nitrogen was applied in two equal splits i.e. 50% at sowing, and 50% at second irrigation (knee height), while P was applied at sowing time. The plant to plant distance was kept 20 cm. Azam variety of maize was sown in June in both years.

To study the effect of soil applied potassium (K) levels (30, 60, 90 kg ha^−1^) and their application time (full at sowing, full at knee height, 50% each at sowing and knee height) along with one control plots (no K applied) on phenology, growth and yield of maize (cv. Azam) under two different field conditions: (i) applied with 5 t ha^−1^ cattle dung and (ii) the second field was without (0 t ha^−1^) cattle dung. The NPK content of the cattle dung was 1.13% N, 0.11% P_2_O_5_, and 0.07% K_2_O. Both the experiments (with and without cattle dung) were conducted under limited irrigation condition (four irrigations) i.e. first at emergence, second at knee height, third at tasseling and fourth at seed fill duration. One irrigation was also applied two weeks before sowing for seedbed preparation. The research was also carried out at the Agronomy Research Farm of The University of Agriculture Peshawar, during summer 2014. The experiment was carried out in randomized complete block (RCB) design with three replications. A plot size of 3 m × 3.5 m was used. Each plot consisted of five rows, 3 m long and 70 cm apart. A uniform dose of 120 kg N ha^−1^ as urea in two equal splits i.e. half at sowing, and half at knee height was applied. Phosphorus was applied at the rate of 30 kg P ha^−1^ as single super phosphate at seedbed preparation. All other agronomic practices were kept uniform and normal for all the treatments. Data were recorded on phenology (days to tasseling, silking and physiological maturity), growth parameters (plant height, glag leaf area, and leaf area index), yield components (ear length, number of grains row^−1^, number of grains ear^−1^, and 1000 grains weight), grain yield and shelling percentage.

Days to tasseling was calculated from the date of sowing to the date when 50% tasseling appeared in each plot. For days to silking, when 50% silking was emerging in each plot, those dates were noted. Days to physiological maturity was recorded from the date of sowing till date when all the plants gets physiological matured (black layer formation) in each plot. Data on plant height (cm) at silking was recorded with the help of meter rod by selecting 10 plants randomly from each plot and then average was worked out. At silking, lengths and widths of flag leaves (most top leaf near tassel) of five plants was measured, then mean flag leaf length and width was calculated. Leaf area index was calculated as leaf area per plant (mean single leaf area × leaves plant^−1^) divided by ground area per plant. Flag leaf area was then calculated using the formula:


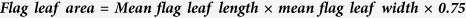


Data on ear length (cm) was recorded with the help of meter rod by selecting ten plants randomly from each plot and then the average was worked out. Data on number of grain rows per ear was calculated by counting grain rows in ten selected ears and then it was averaged. Number of grains ear^−1^ was calculated on ten randomly selected ears from each plot and then average was worked out. Grains weight of randomly 1000 grains was taken from seed lot of each plot and was weighted with the help of electronic balance. Three central rows of each treatment was harvested, dried, threshed, grains were cleaned and weighted and then converted into grain yield (kg ha^−1^). Shelling percentage for each treatment was calculated by using the following formula.


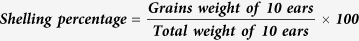


### Statistical Analysis

Data was statistically analyzed according to ref. 31 and means was composed using LSD test (P ≤ 0.05).

## Results

### Response of maize to foliar applied potassium (experiment one)

#### Growth

The physiological maturity (PM) of maize was significantly affected by foliar potassium (K) and application time in year one (2013) as shown in Table 1. However, foliar zinc (Zn) had no significant effects on PM. The rest (all treated plots) took significantly more time to PM (93 days) as compared to control (91 days). Among the foliar K levels, the PM was delayed with 3% K (94 days), while PM was enhanced with the application of 1% K (92 days). Late foliar application at reproductive growth stage delayed the PM (93 days) as compared with early foliar application at vegetative growth (92 days). The PM of maize in year two (2014) was significantly affected control vs. rest, and foliar application time, foliar while K and Zn levels had no significant effects on PM (Table 2). The rest (all treated plots) took significantly more time to PM (95 days) as compared to control (94 days). Late foliar application at reproductive growth stage delayed the PM (95 days) as compared with early foliar application at vegetative growth (94 days).

Foliar K and Zn had significant effect on plant height; however, nutrients application time had no significant effect on plant height of maize in 2013 (Table 1). The rest (all treated plots) had produced significantly taller plants (210 cm) as compared to control (196 cm). Among the foliar K levels, plant height was increased as the K levels were increased. Higher plant heights (214 cm) was obtained with 3% K being at par with 2% K (212 cm), while dwarf plants height (204 cm) was obtained with 1% foliar K. Among the foliar Zn levels, taller plants (212 cm) were obtained with 0.2% Zn and dwarf plants (208 cm) were recorded at 0.3% Zn. Plant height was significantly affected by control vs. rest, foliar K and Zn levels in 2014 (Table 2). The rest (all treated plots) had produced significantly taller plants (194 cm) as compared to control (162 cm). Among the foliar K levels, plant height was increased with 1% foliar K (196 cm) and shorter plants (190 cm) was recorded with 3% K. Among the foliar Zn levels, the highest plant height (197 cm) was obtained with 0.2% Zn, and dwarf plants (191 cm heights) was obtained with 0.1% foliar Zn (191.5 cm).

Foliar K and Zn had significantly affected the mean single leaf area (MSLA), however, the application time had no significant effects on MSLA in 2013 (Table 1). The rest (all treated plots) had significantly larger MSLA (413.3 cm^2^) as compared to control (391.8 cm^2^). In case of foliar K levels, maximum MSLA was obtained with 2% K (417 cm^2^), while lower MSLA was obtained with 1% K (410 cm^2^). Among the foliar Zn levels, higher MSLA was recorded with 0.2% Zn (418 cm^2^) and lower MSLA was recorded with 0.1% Zn (408 cm^2^). The MSLA of maize in 2014 was significantly affected by control vs. rest, foliar K and Zn levels, while application time had no significant effects on MSLA (Table 2).The rest (all treated plots) had produced significantly maximum MSLA (466 cm^2^) as compared to control (377 cm^2^). Among the foliar K levels, maximum MSLA was obtained with 2% K (480 cm^2^) and minimum MSLA was obtained with 1% K (457 cm^2^). Among the foliar Zn levels, maximum MSLA was obtained with 0.2% Zn (485 cm^2^) and minimum MSLA was obtained with 0.1% Zn (449 cm^2^).

Leaf area index (LAI) was significantly affected by control vs. rest, foliar K and Zn levels, while foliar application time was found non-significant on LAI in 2013 (Table 1). The rest (all treated plots) had significantly higher LAI (3.5) as compared to control (3.3) (Table 1). Maximum LAI (3.5 each) was obtained at 2 and 3% K, and lower LAI (3.4) was obtained with 1% K. Among the foliar Zn levels, maximum LAI (3.5 each) was obtained with 0.2 and 0.3% Zn, and lower LAI (3.4) was obtained with 0.1% Zn (Table 1). In 2014, the LAI was significantly affected by control vs. rest, while foliar K and Zn levels and its application time had no significant effects on the LAI of maize (Table 2). The rest (all treated plots) had produced significantly higher LAI (3.2) as compared to control (2.3).

#### Yield and yield components

Data related to biomass yield is given in Table 1 (2013). Statistical analysis of the data demonstrated that foliar K and Zn had significant effect on the biomass yield, while nutrients application time had no significant effect on biomass yield (Table 1). The rest (all treated plots) had produced significantly higher biomass yield (8710 kg ha^−1^) as compared to control (7263 kg ha^−1^). Among the foliar K levels, higher biomass yield was obtained with foliar 3% K (8882 kg ha^−1^) and lower biomass yield was obtained with foliar 1% K (8422 kg ha^−1^). In case of foliar Zn levels, maximum biomass yield was recorded with 0.2% Zn (8977 kg ha^−1^) and minimum biomass yield was recorded with 0.1% foliar Zn (8538 kg ha^−1^). In 2014, the biomass yield of maize was significantly affected by control vs. rest, foliar K and Zn levels, and their application time (Table 2). The rest (all treated plots) had produced significantly higher biomass yield (12840 kg ha^−1^) as compared to control (8968 kg ha^−1^). Among the K levels, the highest biomass yield was obtained with 3% foliar K (13690 kg ha^−1^) and the lowest yield was obtained with 2% K (12249 kg ha^−1^). Among the foliar Zn levels, the highest biomass yield was obtained with 0.1% Zn (13426 kg ha^−1^) and the lowest yield was obtained with 0.2% Zn (12037 kg ha^−1^). Early foliar spray at vegetative growth stages resulted in higher biomass (13924 kg ha^−1^) than late spray at the reproductive growth stage (11755 kg ha^−1^).

Statistical analysis of the data revealed that only control vs. rest had significant effect on thousand grains weight (TGW) of maize in 2013 (Table 3). Foliar K levels, Zn levels, K × Zn and application time had no significant effects on TGW of maize. The rest (all treated plots) had produced significantly higher TGW (213 g) as compared to control plot (206 g). The TGW in year two was significantly affected by control vs. rest, foliar K and Zn levels, and K × Zn, while application time had no significant effect on TGW of maize in year two (Table 4). The rest (all treated plots) had produced significantly higher TGW (224 g) as compared to control (183 g). Among foliar K levels, the maximum TGW was obtained with 2% K (228 g), while minimum TGW was obtained with 3% K (217 g). Among foliar Zn levels, application of 0.2% foliar Zn gave maximum TGW (229 g), while 0.1% foliar Zn gave minimum TGW (218 g).

Foliar K and Zn levels had significant effects, while nutrients application time had no significant effect on number of grains ear^−1^ in 2013 (Table 3). The rest (all treated plots) had produced significantly more number of grains ear^−1^ (397) as compared to control plot (362). In case of foliar K levels, the maximum number of grains ear^−1^ (402) was obtained with 2% K being statistically the same with 3% K (401). However, minimum number of grains ear^−1^ (389) was recorded with 1% K. Among foliar Zn levels, maximum number of grains ear^−1^ (406) was obtained with 0.2% Zn, while minimum number of grains ear^−1^ (391) was recorded with 0.1% Zn. Number of grains ear^−1^ in year two was significantly affected by control vs. rest, foliar K and Zn levels, and their application time (Table 4). The rest (all treated plots) had produced significantly higher number of grains ear^−1^ (456) as compared to control (321). Among the foliar K levels, maximum number of grains ear^−1^ was obtained with 2% foliar K (478) and minimum (430) was obtained with 3% K. among the Zn levels, maximum grains ear^−1^ was obtained with 0.2% Zn (499) and minimum (412) was obtained with 0.3% foliar Zn. Early foliar spray at vegetative growth stages resulted in lower number of grains ear^−1^ (442) than late spray at the reproductive growth stage (471).

Grain yield was significantly affected by foliar K and Zn levels in 2013 (Table 3). However, their application time had no significant effect on grain yield of maize. The rest (all treated plots) had produced significantly higher grain yield (3543 kg ha^−1^) as compared to control (2509 kg ha^−1^). In case of the foliar K levels, maximum grain yield (3778 kg ha^−1^) was obtained with 3% K being at par with 2% K (3677 kg ha^−1^). However, minimum grain yield (3175 kg ha^−1^) was obtained with 1% foliar K. In case of foliar Zn levels, higher grain yield (3717 kg ha^−1^) was recorded in plots with 0.2% Zn, and lower grain yield (3455 kg ha^−1^) was obtained with foliar 0.3% Zn. Grain yield of maize was significantly affected by control vs. rest, foliar K and Zn levels, application time and K × Zn in year 2014 (Table 4). The rest (all treated plots) had produced significantly higher grain yield (3038 kg ha^1^) than control (2341 kg ha^−1^). Among the foliar K levels, the highest grain yield was obtained with 2% K (3241 kg ha^−1^), while the lowest grain yield was obtained with 1% K (2804 kg ha^−1^). Among the foliar Zn levels, the highest grain yield was obtained with 0.2% Zn (3280 kg ha^−1^), while the lowest grain yield was obtained with 0.3% Zn (2791 kg ha^−1^). Early foliar spray at vegetative growth stages resulted in higher grain yield (3272 kg ha^−1^) than late spray at the reproductive growth stage (2804 kg ha^−1^).

Foliar K and Zn had significant effect on the shelling percentage, while K × Zn and application time had no significant effect in 2013 (Table 3). The rest (all treated plots) had significantly higher shelling (82%) as compared to control (79%). In case of the foliar K levels, maximum shelling (83%) was obtained with 3% K, whereas lower shelling (82%) was recorded in plots treated with each 1 and 2% foliar K. In case of foliar Zn levels, maximum shelling (83%) was calculated for 0.2% Zn. Application of each 0.1 and 0.3% foliar Zn had less shelling (82%). Shelling percentage in 2014 was significantly affected by control vs. rest, foliar K and Zn levels, while application time had no significant effects on shelling percentage (Table 4). The rest (all treated plots) had significantly higher shelling (82%) as compared to control (77%). In case of foliar K levels, the highest shelling percentage was obtained with 1% foliar K (84%), while the lowest shelling percentage was obtained with 3% K (80%). Among the Zn levels, the highest shelling percentage was obtained with 0.2% Zn (84) and the lowest shelling percentage was obtained with 0.1% Zn (78%).

Statistical analysis of the data indicated that foliar K had significant effect on the harvest index, while foliar Zn, and application time had no significant effect on harvest index in maize in 2013 (Table 3). The rest (all treated plots) had significantly higher harvest index (40%) as compared to control (33%). In case of the foliar K levels, higher harvest index was recorded for the plots treated with 3% foliar K (42%) and the lowest harvest index was noted for the plots sprayed with 1% foliar K (39%). Harvest index was significantly affected by control vs. rest, foliar K and Zn levels, while application time had no significant effects on harvest index of maize in 2014 (Table 4). The rest (all treated plots) had significantly higher harvest index (24%) as compared to control (18%). Among the foliar K levels, the highest harvest index was obtained with 2% K (27%) and the lowest harvest index was obtained with 1% K (23%). Among the foliar Zn levels, the highest harvest index were obtained with 0.2% Zn (28%) and the lowest harvest index was calculated for 0.3% Zn (22%).

### Response of maize to soil applied potassium (experiment two)

#### Growth

Statistical analysis of the data exhibited that days to tasseling of maize was significantly affected by control vs. rest (treated plots), cattle manure, K levels and K application time (Table 5). The treated plots (rest) had took minimum of 56 days to tasseling than control (57 days). Minimum of 55 days to tasseling was recorded from the field under cattle manure as compared to without cattle manure (56 days). Tasseling was delayed to 57 days when K was applied at the low rate of 30 kg K ha^−1^, followed by 56 days with 60 kg K ha^1^, while earlier days to tasseling (55 days) was observed when K was applied at the highest rate of 90 kg K ha^−1^. Days to tasseling was delayed (56 days) when 100% K was applied at knee height, while earlier tasseling (55 days) was observed when 100% K was applied at the sowing time.

Days to silking was significantly affected by control vs. rest, cattle manure, K levels and K application timings (Table 5). The treated plots (rest) took less number of 62 days to silking than control which took 64 days to tasseling. Earlier silking (62 days) was recorded from the field under cattle manure as compared to the field without cattle manure (63 days). Silking was delayed to 63 days when K was applied at the low rates (30 kg K ha^−1^ and 60 kg K ha^1^), while earlier silking (62 days) was recorded when K was applied at the highest rate (90 kg ha^−1^). Silking was delayed (63 days) when K was applied in two equal splits, while earlier silking (55 days) was recorded in those plots when 100% K was applied at sowing time.

Physiological maturity was significantly affected by control vs. rest, cattle manure, K levels and K application timing (Table 5). The treated plots (rest) had earlier physiological maturity (93 days) than control which take 95 days. Earlier days to physiological maturity (92 days) was recorded under cattle manure as compared to without cattle manure field (93 days). Delayed physiological maturity (93 days) was recorded when K was applied at the low rates of 30 kg K ha^−1^ and 60 kg ha^1^ as compared with earlier physiological maturity (92 days) when K was applied at the highest rate (90 kg ha^−1^). Physiological maturity was delayed to 93 days when K was applied in two equal splits and earlier physiological maturity (92 days) was recorded in those plots when 100% K was applied at sowing time (Table 5).

Plant height of maize was significantly affected by control vs. rest, cattle manure, K levels and K application timing (Table 5). The treated plots (rest) had produced significantly taller plants (216 cm height) than control with 199 cm plant height (Table 5). Tallest plants (222.09 cm) were recorded from the cattle manure experiment as compared to without cattle manure experiment (208.8 cm). Tallest plants (223.7 cm height) were recorded for the plots treated with the highest K level (90 kg ha^−1^), followed by (215.8 cm) with 60 kg K ha^−1^, while dwarf plants of 208.0 cm heights were recorded for the plots that received only 30 kg K ha^−1^. Tallest plants (220.1 cm) were recorded for the plots which received K in two equal splits (50% K at sowing time and 50% K at knee height), followed by (216.4 cm) in those plots which received 100% K at sowing time, while dwarf plants of 211.0 cm heights were recorded for the plots which received 100% K at knee height (Table 5).

Flag leaf area (cm^2^) of maize was significantly affected by control vs. rest, cattle manure, K levels and K application timing (Table 5). The treated plots (rest) had significantly maximum flag leaf area of 117 cm^2^ than control (98 cm^2^). The maximum flag leaf area (127.6 cm^2^) was recorded from the experiment under cattle manure as compared to without cattle manure (105.9 cm^2^). Maximum flag leaf area (120.6 cm^2^) was recorded for the plots treated with the highest K level (90 kg ha^−1^), followed by (116.5 cm^2^) with 60 kg K ha^−1^, while minimum flag leaf area of 113.3 cm^2^ was recorded for the plots that received the low rate of 30 kg K ha^−1^. The highest flag leaf area (120.3 cm^2^) was recorded for the plots which received 50% K at sowing time +50% K at knee height, followed by 118.3 cm^2^ (100% K at sowing time), while the lowest flag leaf area of 111.8 cm^2^ was recorded from the plots which received 100% K at knee height (Table 5).

Leaf area index of maize was significantly affected by control vs. rest, cattle manure, K levels and K application timing (Table 5). The treated plots (rest) had significantly maximum leaf area index (2.9) than control (2.6). The maximum leaf area index (3.2) was recorded from the cattle manure experiment as compared to without cattle manure (2.7). Maximum leaf area index (3.1) was recorded for the plots treated with the highest K level (90 kg ha^−1^), followed by 2.9 with 60 kg K ha^−1^, while the lowest leaf area index (2.7) was recorded from the plots that received 30 kg K ha^−1^. The highest leaf area index (3.1) was recorded for the plots which received 50% K at sowing time +50% K at knee height, while application 100% K at sowing time and 100% K at knee height resulted lower leaf area index of 2.9 and 2.8, respectively.

#### Yield and yield components

Ear length was significantly affected by control vs. rest, cattle manure, K levels and K application timing (Table 6). The treated plots (rest) had significantly maximum ear length (16.2 cm) than control (14.0 cm). The maximum ear length (16.5 cm) was recorded from the cattle manure field and 15.9 cm ear length was recorded for the experiment without cattle manure being statistically at par with each other. Among the K levels, the maximum ear length (16.7 cm) was recorded for the plots treated with the highest K level (90 kg ha^−1^) and 16.2 cm length with 60 kg K ha^−1^ which were statistically par with each other, while minimum ear length of 15.7 cm was recorded from the plots that received the lowest rate of 30 kg K ha^−1^. Among the K application timing, the maximum ear lengths (16.6 cm) and (16.1 cm) was recorded for the plots which received K in two equal splits (50% K at sowing time +50% K at knee height) and 100% K at sowing time, respectively which were statistically par with each other. The minimum ear length of 15.9 cm was recorded from the plots which received 100% K at knee height (Table 6).

Number of grains row^−1^ of maize was significantly affected by control vs. rest, cattle manure, K levels and K application timing (Table 6). The treated plots (rest) had significantly maximum number of grains (35) than control (28) as shown in Table 6. The highest number of grains (36) was obtained from the field under cattle manure as compared to the field without cattle manure (34). Among the K levels, the highest number of grains (37) was obtained from the plots treated with the highest K level (90 kg ha^−1^), followed by (35) with 60 kg K ha^−1^, while the lowest number of grains row^−1^ (34) was obtained from the plots that received 30 kg K ha^−1^. Among the K application timing, the highest number of grains (36) was recorded for the plots which received 50% K at sowing time +50% K at knee height, followed by 35 grains row^−1^ those plots which received 100% K at sowing time, while the lowest number of grains (34) was obtained from the plots which received 100% K at knee height.

Number of grains ear^−1^ of maize was significantly affected by control vs. rest, cattle manure, K levels and K application timing (Table 6). The treated plots (Table 6) had significantly higher grains ear^−1^ (453) than control (383). The highest number of grains ear^−1^ (474.1) was obtained from the cattle manure field as compared to without cattle manure (431.5). Among the K levels, the highest grains ear^−1^ (468.2) was obtained from the plots treated with the highest K level (90 kg ha^−1^), followed by 451.6 with 60 kg K ha^−1^, while the lowest grains ear^−1^ (438.6) was obtained from the plots that received 30 kg K ha^−1^. Among the K application timing, the highest grains ear^−1^ (478.8) was recorded for the plots which received 50% K at sowing time +50% K at knee height, while K application full at sowing resulted in the lower number of grains ear^−1^ (445.4) which was at par with 100% K application at knee height (434.2).

Thousand grains weight (g) of maize was significantly affected by control vs. rest, cattle manure, K levels and K application timing (Table 6). The treated plots (rest) had significantly higher thousand grains weight (256 g) than control (226.7 g). The highest thousand grains weight (261.3 g) was obtained from the cattle manure field as compared to the field without cattle manure (250.6 g). Among the K levels, the highest thousand grains weight (261.8 g) was recorded for the plots treated with the highest K level (90 kg ha^−1^). K application at the rate of 60 kg ha^−1^ and 30 kg ha^−1^ resulted in lower thousand grain weights of 255.2 g and 250.9 g, respectively. Among the K application timing, the highest thousand grain weight (259.6 g) was recorded for the plots which received 50% K at sowing time +50% K at knee height, followed by in those plots which received 100% K at sowing time (257.1 g) which was at par with 100% K at knee height (251.3 g/1000 grains).

Grain yield of maize was significantly affected by control vs. rest, cattle manure, K levels and application timing (Table 6). The treated plots (rest) had significantly higher grain yield (4307 kg ha^−1^) than control (3709 kg ha^−1^) (Table 6). The experiment under cattle manure produced higher grain yield (4507 kg ha^−1^) than without cattle manure (4107 kg ha^−1^). Among the K levels, the highest grain yield (4612 kg ha^−1^) was recorded for the plots treated with the highest K level (90 kg ha^−1^), followed by 4364 kg ha^−1^ with 60 kg K ha^−1^, while the lowest grain yield (3945 kg ha^−1^) was obtained from the plots that received 30 kg K ha^−1^. Among the K application timing, the highest grain yield (4407 kg ha^−1^) was recorded for the plots which received the required K in two equal splits i.e. 50% at sowing and 50% at knee height. The lowest grain yield (4174 kg ha^−1^) was obtained from the plots which received 100% K at knee height in one split only.

Shelling percentage of maize was significantly affected by control vs. rest, cattle manure and K levels (Table 6). The treated plots (rest) had significantly higher shelling percentage (78.6%) than control having 73.7% shelling (Table 6). The highest shelling percentage (79.6%) was obtained from the experiment under cattle manure as compared to without cattle manure (77.6%). Among the K levels, the highest shelling percentage (79.6%) was obtained from the plots treated with the highest K level (90 kg ha^−1^) which was at par with 60 kg K ha^−1^ (79.3%), while the lowest shelling percentage (76.9%) was obtained from the plots that received 30 kg K ha^−1^.

## Discussion

### Response of maize to foliar applied potassium (experiment one)

The PM (physiological maturity) was significantly delayed in the rest (all treated plots) as compared to control (no spray) in both experimental years. In year one (2013), the PM was delayed with increase in the level of foliar K, while in year two (2014), foliar K had no significant effect on PM of maize. Application of K increased phosphorus and nitrogen uptake, which led to luxurious vegetative growth^32^,^33^ reported that soil K application delay PM in maize. In both years, foliar Zn showed no significant effect on PM. Late foliar application at reproductive growth stage delayed the PM as compared with early foliar application at vegetative growth in both years^34^ suggested that K application enhanced silking in maize, but lengthens grain fill duration and therefore increased grain yield potential. Delayed silking and physiological maturity was observed in maize when foliar nitrogen was sprayed in the form of urea and calcium ammonium nitrate, while foliar application of ammonium sulphate enhanced phenological development in maize^21^. They^21^,^30^ reported that late application of foliar-N increased the vegetative growth period, resulting in delayed tasseling, silking and physiological maturity.

The rest of the treated plots had produced significantly taller plants than control in both years. The increase in plant height with foliar K and Zn probably may be due to the activation of enzymes that helped the plants to increase their heights. Plant height was increased as the foliar K levels were increased in 2013, while in 2014; plant height was decreased with increase in foliar K level. According to ref. 35, plant heights increased with K application. Increase in Zn levels resulted in dwarf plants in 2013, while in 2014 dwarf plants were noted with lowest Zn levels^36^,^37^,^38^ reported that higher plant height was obtained in maize with foliar Zn application^39^ reported that under Zn deficiency shortening of the internodes and reduction in growth occurred. Our results in both years confirmed that foliar application time had no significant impact on the heights of maize plants. However, our earlier studies^21^,^30^ regarding foliar N application at various growth stages of maize revealed that late application of foliar N increased plant height in maize.

The MSLA and LAI were higher in the rest of the foliar treated plots than control in both years. The increase in MSLA and LAI with foliar K and Zn could be due to the increased photosynthetic activity of plants^39^ that Zn deficiency cause symptoms of strong chlorosis and decrease of the leaf production besides the reduction of crop growth. In both years, the maximum MSLA and LAI was obtained with 2 and 3% K; while lower MSLA and LAI was obtained with 1% K^32^ reported significant increase in leaf area with the application of K. Similarly, the MSLA was higher with 0.2% Zn and lower MSLA was recorded with 0.1% Zn in both years. Our results in both years confirmed that foliar application time had no significant impact on the MSLA of maize plants. However, our earlier research^30^ indicated that MSLA in maize was significantly affected with the application times of foliar nitrogen in maize.

The rest of the foliar treated plots had produced significantly higher TGW over control in both years. The reason for higher thousand grain weight in the rest of foliar treated plots probably might be due to the increased photosynthetic activity of plants which finally moved to the sink and produce high weighted grains^32^. In 2013, foliar K had no significant effect on TGW; while in 2014; maximum TGW was obtained with 2% K and minimum with 3% K. In 2013, foliar Zn had no significant effect on TGW; while in 2014, application of 0.2% foliar Zn gave maximum and 0.1% gave minimum TGW. Our results in both years confirmed that foliar application time had no significant impact on TGW of maize.

The rest of the foliar treated plots had produced significantly more number of grains ear^−1^ over control in both years. In 2013, more number of grains ear^−1^ was obtained with 2 and 3% than 1% foliar K, while in 2014, maximum number of grains ear^−1^ was also obtained with 2% foliar K but minimum with 3% K^35^ found that grains ear^−1^ was increased with the application of K. In 2013, number of grains ear^−1^ increased with 0.2% and minimum with 0.1% foliar Zn, while in 2014, maximum grains ear^−1^ was obtained with 0.2% Zn and minimum with 0.3% foliar Zn^38^ reported that Zn application increased the number of grains ear^−1^ in maize. In 2013, foliar application time had no significant effect on number of grains ear^−1^, while in 2014, late foliar spray at reproductive growth stage resulted in more number of grains ear^−1^ than early spray at the vegetative growth stage. According to^26^, foliar application of K at grain filling stage was more effective in alleviating the adverse effect of water deficit on number of spikelets per spike, 1000-grains weight and grain yield and improved these by 8.76, 35.84 and 49.03%, respectively, in wheat crop.

The rest of the foliar treated plots had produced significantly higher biomass yield over control in both years. The increase in biomass yield in the foliar treated plots was attributed to the increase in plant heights, greater MSLA and LAI and higher number of grains ear^−1^ and heavy grains^39^ reported that Zn deficiency cause symptoms of strong chlorosis, decrease of the leaf production, besides the reduction of growth and yield. In 2013, higher biomass was obtained with foliar 3% K and lower biomass yield was obtained with foliar 1% K. In 2014, the highest biomass yield was also obtained with 3% foliar K but the lowest yield was obtained with 2%. In 2013, maximum biomass was obtained with 0.2% Zn and minimum with 0.1% foliar Zn. In 2014, the highest biomass yield was obtained with 0.1% Zn but lowest biomass yield was obtained with 0.2% Zn. In 2013, foliar application time had no significant effects on biomass yield^38^ noted that biomass yield varied significantly with different foliar Zn levels. In 2014, early foliar spray at vegetative growth stages resulted in higher biomass than late spray at the reproductive growth stage.

The rest of the foliar treated plots had produced significantly higher grain yield over control in both years. The increase in grain yield in the foliar treated plots was attributed to the increase yield components (number of grains ear^−1^ and 1000 grains weight), increase in shelling percentage and harvest index. Grain yield reduced by 22.6–26.4% due to decrease in kernel number and weight^40^, and decreased by 37% due to a decline of 18% in kernel weight and 10% in kernel number under dryland conditions^41^. In both years, grain yield was higher with 2 and 3% foliar K than 1% foliar K^42^ reported that K application increased the grain yield in maize. In both years, higher grain yield was obtained with 0.2% Zn and minimum with 0.3% foliar Zn^43^ reported that Zn application increased the grain yield of maize. According to ref. 44, application of Zn in the soil promoted a higher Zn uptake by the plants and maize yield, compared to the application via seeds or foliar. In 2013, foliar application time had no significant effects on grain yield. In 2014, the early foliar spray at vegetative growth stage resulted in higher grain yield than late spray at the reproductive growth stage^45^ reported yield increases of 27–31% when N-P-K-S fertilizer was sprayed at late reproductive stages (R5 to R6) in soybean^26^ reported that foliar application of K at grain filling stage of wheat alleviated the adverse effect of water deficit and increased the yield and yield components tremendously.

The rest (all treated plots) had significantly higher shelling percentage over control in both years. The increase in shelling percentage in the foliar treated plots was attributed to the increase in number of grains ear^−1^ and 1000 grains weight^30^ reported increase in shelling percentage with foliar nitrogen by improving the yield contributing characters. In 2013, shelling percentage was increased with 3% foliar K than 1 and 2% foliar K. In 2014, the highest shelling percentage was obtained with 1% foliar K while the lowest shelling percentage was obtained with 3% foliar K. In both years, maximum shelling was calculated for 0.2% foliar Zn than 0.1 and 0.3% foliar Zn. Our results in both years confirmed that foliar application time had no significant impact on the shelling percentage of maize.

The rest of the treated plots had significantly higher harvest index over control in both years. The increase in harvest index in the foliar treated plots was attributed to the increase in yield components and grain yield. In 2013, higher harvest index was recorded for the plots treated with 3% foliar K and the lowest harvest index was noted for the plots sprayed with 1% foliar K. In 2014, the highest harvest index was obtained with 2% K and the lowest harvest index was obtained with 1% foliar K. In 2013, the foliar Zn levels have no significant effect on harvest index of maize. In 2014, the highest harvest index was obtained with 0.2% Zn (and the lowest with 0.3% foliar Zn. Our results in both years confirmed that foliar application time had no significant impact on the harvest index of maize^30^ reported that increase in yield and yield components with foliar urea spray increased harvest index in maize^21^ reported that foliar N sources had significant effects, while application time had no significant effects on the harvest index of maize.

### Response of maize to soil applied potassium (experiment two)

Phenological development (days to tasseling, silking and physiological maturity) was delayed with lower rate of potash (30 kg K ha^−1^) and application of K in two equal splits. Earlier phenological development in maize was observed with the application of K at higher rates (90 kg ha^−1^) and full application at sowing time^46^,^47^ reported that increase in number of K splits delayed phenological parameters, while increase in K level up to 60 kg ha^−1^ delayed phenological parameters but further increase in K level up to 90 kg ha^−1^ enhanced phenological parameters. But according to ref. 48, days to tasseling remained unaffected by increase in K level^33^ reported that days to 50% silking delayed with the application of higher dose of K^49^ reported that manure application affect growth rate of maize; application of manure resulted in earlier days to tasseling, silking and maturity.

Application of potash at the highest rate (90 kg ha^−1^), and application of K in two equal splits, and application of cattle manure improved growth parameters (plant height, leaf area and number, and leaf area index)^10^,^33^ reported that the main reason for improved growth parameters at higher K levels could be due to the activation of several enzymes, increase in protein synthesis, N uptake and utilization that results in the normal growth and hence maize attained maximum growth. Saleem *et al.* (2011) reported that single application of K at the time of sowing did not provide sufficient amount of K that required for the plants at the later growth stages and hence the plant height and leaf area of the maize plants not increased. However^46^,^48^, reported that different K doses could not affect plant growth in maize^50^,^51^,^52^,^53^ reported that application of poultry manure increase plant growth because more nutrients were made readily available and easily absorbable by receiving plants leading to faster growth and development^54^ reported taller plants and more number of leaves of pepper resulting from application of higher rate of poultry manure^35^ reported that plant height in maize increased with the application of K as compared to control.

Yield components (ear length, grains row^−1^, grains ear^−1^ and thousand grains weight), grain yield and shelling percentage was increased when K was applied at the highest rate of 90 kg ha^−1^, K applied in two equal splits, and when applied with cattle manure. The reason for higher yield and yield components with increase in K level and in two equal splits, and cattle manure application probably may be attributed to the maximum availability of K that may have increased photosynthetic activities and more dry matter was accumulated and partitioned to the grains (data not shown)^55^ reported that increase in yield components with K fertilization might be due to K has important role in improving water use efficiency, improved plant growth, increase cell division and also results in quick transportation of assimilates towards grains. The reason for highest thousand grain weight with K application could be that K increased the photosynthetic activity of plants^32^ which finally moved to the sink and produce highest weighted grains^46^ reported that maize produced maximum number of grains ear^−1^ and increased grains weight of maize with increase in K level^10^ reported that K application increases the number of grains ear^−1^ due to the improved activity of enzymes in the plant leading to more intensive assimilation and translocation of assimilates from the leaf to the grains^56^,^57^ reported significant increase in grains weight of maize with increase in K level^58^,^59^,^60^ found that the number of grains ear^−1^ of maize obtained from plants that received poultry manure. According to ref. 61, the higher 1000 seeds weight was due to the large accumulation of proteins and other reserved food in the seeds, was attributed to the increase in the availability of nutrients when chemical fertilizers were applied mixed with organic manures.

Grain yield was significantly increased while increasing K levels, number of K splits and application of cattle manure. The increase in grain yield with increase in K level, number of K splits and application of cattle manure was attributed to the delay in phenological development, higher flag leaf area and leaf area index, and more yield components^62^ reported that the probable reason for increase in grain yield and was due to role of K increased the rate of CO_2_ assimilation, stabilized the stomata regulation, improved stomata closure and enzyme activity as a result of which more carbohydrates might have produced which might have increased grain yield^48^,^63^ also found a significant increase in yield of maize with the application of K over the control plots^64^,^65^ suggested that increasing rates of manures positively affected maize grain yield. The increase in grain yield with poultry manure was mainly due to more ear lengths, ear diameter as well as more number of grains ear^−1^ and better grain development. The increase in shelling percentage with treated plots than control, cattle manure and higher K level was attributed to the increase in grains weight and more number of grains per ear^66^.

## Conclusions

It was concluded from experiment one (foliar applied K) that foliar K application improve growth, increased yield and yield components of dryland maize under semiarid climates. Application of 1 to 3% foliar K and 0.1 to 0.2% foliar Zn was found most beneficial in terms of better growth, higher yield and yield components of maize than control (no foliar spray). Foliar fertilization of field crops with macro and micro nutrients provides food (essential nutrients) and also supplements the low and unreliable rainfall under semiarid climates. On the basis of our two yeas results obtained, we hypothesize that this kind of foliar fertilization could also be beneficial for other crops in similar semiarid climates. From the second experiment (soil applied K), we concluded from this study that application of K at the higher rate of 90 kg K ha^−1^ in two equal splits (50% at sowing and 50% at knee height) along with cattle dung (5 t ha^−1^) improve maize growth characters, increase yield and yield components as well as growers income under limited irrigation condition.

## Additional Information

**How to cite this article**: Amanullah *et al.* Potassium Management for Improving Growth and Grain Yield of Maize (*Zea mays* L.) under Moisture Stress Condition. *Sci. Rep.* **6**, 34627; doi: 10.1038/srep34627 (2016).

## Figures and Tables

**Table 1:** Days to physiological maturity, plant height, mean single leaf area, leaf area index, and biomass yield as affected by foliar K, Zn and their application time in year one (2013).

	Physiological maturity (days)	Plant height (cm)	Mean single leaf area (cm^−2^)	Leaf area index	Biomass yield (kg ha^−1^)
Foliar K (%)					
1	92 c	204 b	410 b	3.4 b	8422 b
2	93 b	212 a	417 a	3.5 a	8826 a
3	94 a	214 a	413 b	3.5 a	8882 a
**LSD**	**0.4**	**3.3**	**3.7**	**0.03**	**296**
Foliar Zn (%)					
0.1	93 a	209 b	408 c	3.4 b	8538 b
0.2	93 a	212 a	418 a	3.5 a	8977 a
0.3	93 a	208 b	414 b	3.5 a	8614 b
**LSD**	**ns**	**3.1**	**3.5**	**0.03**	**296**
Application time					
Vegetative	92 b	207 a	409 a	3.4 a	8297 a
Reproductive	93 a	206 a	408 a	3.5 a	8399 a
** Significance**	*	**ns**	**ns**	**ns**	**ns**
Control vs Rest					
Control	91b	196 b	392 b	3.3 b	7263 b
Rest	93 a	210 a	413 a	3.5 a	8710 a
**Significance**	**	**	**	**	**

Means followed by the same letters within same category in the same columns are not different statistically.

^*^Significant at P ≤ 0.05.

^**^Significant at P ≤ 0.01.

ns = non-significant at P ≤ 0.05.

**Table 2:** Days to physiological maturity, plant height, mean single leaf area, leaf area index, and biomass yield as affected by foliar K, Zn and their application time in year two (2014).

	Physiological maturity (days)	Plant height (cm)	Mean single leaf area (cm^−2^)	Leaf area index	Biomass yield (kg ha^−1^)
Foliar K (%)					
1	95	196 a	457	2.7 a	12579 b
2	95	195 ab	480	2.9 a	12249 c
3	95	190 b	459	2.9 a	13690 a
**LSD**	**ns**	**4.7**	**20**	**ns**	**1139**
Foliar Zn (%)					
0.1	95	191 b	449	2.6 a	13426 a
0.2	95	197 a	485	2.8 a	12037 b
0.3	95	192 b	461	3.1 a	13056 ab
**LSD**	**ns**	**4.7**	**20**		**1139**
Application time					
Vegetative	94	193 a	467	2.7 a	13924 a
Reproductive	95	194 a	463	3.0 a	11755 b
**Significance**	*	**ns**	**ns**	**ns**	*
Control vs Rest					
Control	94	162 b	377 b	2.3 b	8968 b
Rest	95	194 a	466 a	3.2 a	12840 a
**Significance**	*	**	**	*	**

Means followed by the same letters within same category in the same columns are not different statistically.

^*^Significant at P ≤ 0.05.

^**^Significant at P ≤ 0.01.

ns = non-significant at P ≤ 0.05.

**Table 3:** Thousand grains weight, grains ear^−1^, grain yield, shelling percentage, and harvest index of maize as affected by foliar K, Zn and their application time in year one (2013).

	1000 grains weight (g)	Number of grains ear^−1^	Grain yield (kg ha^−1^)	Shelling percentage	Harvest Index (%)
Foliar K (%)					
1	211 a	389 b	3175 b	82 b	39 b
2	214 a	402 a	3677 a	82 b	41 a
3	215 a	401 a	3778 a	83 a	42 a
**LSD**	**ns**	**3.6**	**184**	**0.5**	**1.8**
Foliar Zn (%)					
0.1	211 a	391 b	3458 b	82 b	40 a
0.2	216 a	406 a	3717 a	83 a	41 a
0.3	212 a	394 b	3455 b	82 b	40 a
**LSD**	**ns**	**3.9**	**159**	**0.7**	**ns**
Application time					
Vegetative	212 a	390 a	3247 a	81 a	38 a
Reproductive	211 a	387 a	3322 a	82 a	39 a
**Significance**	**ns**	**ns**	**ns**	**ns**	**ns**
Control vs Rest					
Control	206 b	362 b	2509 b	79 b	33 b
Rest	213 a	397 a	3543 a	82 a	40 a
**Significance**	*	**	**	**	**

Means followed by the same letters within same category in the same columns are not different statistically.

^*^Significant at P ≤ 0.05.

^**^Significant at P ≤ 0.01.

ns = non-significant at P ≤ 0.05.

**Table 4:** Thousand grains weight, grains ear^−1^, grain yield, shelling percentage, and harvest index of maize as affected by foliar K, Zn and their application time in year two (2014).

	1000 grains weight (g)	Number of grains ear^−1^	Grain yield (kg ha^−1^)	Shelling percentage	Harvest Index (%)
Foliar K (%)					
1	225 a	461a	2804 c	84 a	23 b
2	228 a	478 a	3241a	82 b	27 a
3	217 b	430 b	3069 b	80 c	23 b
**LSD**	**8.4**	**8.4**	**339**	**2**	**3.5**
Foliar Zn (%)					
0.1	218 b	458 b	3042 b	78 b	23 b
0.2	229 a	499 a	3280 a	84 a	28 a
0.3	222 b	412 c	2791c	84 a	22 b
**LSD**	**8.4**	**8.4**	**339**	**2**	**3.5**
Application time					
Vegetative	222 a	442 b	3272 a	82 a	24 a
Reproductive	224 a	471 a	2804 b	82 a	24 a
**Significance**	**ns**	*	*	**ns**	**ns**
Control vs Rest					
Control	183 b	321 b	2341 b	77 b	18 b
Rest	224 a	456 a	3038 a	82 a	24 a
**Significance**	**	**	**	*	**

Means followed by the same letters within same category in the same columns are not different statistically.

^*^Significant at P ≤ 0.05.

^**^Significant at P ≤ 0.01.

ns = non-significant at P ≤ 0.05.

**Table 5:** Days to tasseling, silking and physiological maturity, plant height, flag leaf area and leaf area index of maize as affected by soil applied potassium (K) levels (kg ha^−1^) and its application time with and without cattle dung.

Treatments	Days to tasseling	Days to silking	Days to physiological maturity	Plant height (cm)	Flag leaf area (cm^−2^)	Leaf area index
30 kg K ha^−1^	57 a	63 a	93 a	208.0 c	113.3 b	2.7 c
60 kg K ha^−1^	56 b	63 a	93 a	215.8 b	116.5 ab	2.9 b
90 kg K ha^−1^	55 c	62 b	92 b	223.7 a	120.6 a	3.1 a
100% K at sowing	55 b	62 b	92 b	216.4 ab	118.3 a	2.9 b
100% K at knee height	56 a	62 b	93 a	211.0 b	111.8 b	2.8 b
50% K each at sowing and knee height	56 a	63 a	93 a	220.1 a	120.3a	3.1 a
With cattle dung	55 b	62 b	92 b	222.9 a	127.6 a	3.2 a
Without cattle dung	56 a	63 a	94 a	208.8 b	105.9 b	2.7 b
Control plots	57 a	64 a	95 a	199.1 b	98.2 b	2.6 a
Treated (rest) plots	56 b	62 b	93 b	216.3 a	117.5 a	2.9 a

Means of the same category followed by different letters are significantly different from each other using LSD test (P ≤ 0.05).

**Table 6:** Ear length, number of rows and grains ear^−1^, 1000 grains weight, grain yield and shelling percentage of maize as affected by soil applied potassium (K) levels (kg ha^−1^) and its application time with and without cattle dung.

Treatments	Ear length (cm)	Rows ear^−1^ (number)	Grains ear^−1^ (number)	1000 grains weight (g)	Grain yield (kg ha^−1^)	Shelling (%)
30 kg K ha^−1^	15.7 b	34 c	438.6 b	250.9 b	3945 c	76.9 b
60 kg K ha^−1^	16.2 ab	35 b	451.6 ab	255.2 b	4364 b	79.3 a
90 kg K ha^−1^	16.7 a	37 a	468.2 a	261.8 a	4612 a	79.6 a
100% K at sowing	16.1 ab	35 b	445.4 b	257.1 ab	4340 b	79.7 a
100% K at knee height	15.9 b	34 c	434.2 b	251.3 b	4174 c	78.3 a
50% K each at sowing and knee height	16.6 a	36 a	478.8 a	259.6 a	4407 a	78.2 a
With cattle dung	16.5 a	36 a	474.1 a	261.3 a	4507 a	79.6 b
Without cattle dung	15.9 ab	34 b	431.5 b	250.6 b	4107 b	77.6 a
Control plots	14.0 b	28 b	383 b	226.7 b	3709 b	73.7 b
Treated (rest) plots	16.2 a	35 a	453 a	256.0 a	4307 a	78.6 a

Means of the same category followed by different letters are significantly different from each other using LSD test (P ≤ 0.05).
